# Clin-JEPA: A Multi-Phase Co-Training Framework for Joint-Embedding Predictive Pretraining on EHR Patient Trajectories

**Published:** 2026-07-04

**Authors:** Yixuan Yang, Mehak Arora, Ryan Zhang, Baraa Abed, Junseob Kim, Tilendra Choudhary, Md Hassanuzzaman, Kevin Zhu, Ayman Ali, Chengkun Yang, Alasdair Edward Gent, Victor Moas, Rishikesan Kamaleswaran

**Affiliations:** Duke University, Durham, NC, USA

## Abstract

We present Clin-JEPA, a multi-phase co-training framework for joint-embedding predictive (JEPA) pretraining on electronic health record (EHR) patient trajectories. JEPA architectures have enabled latent-space planning in robotics and high-quality representation learning in vision, but extending the paradigm to EHR data—to obtain a single backbone that simultaneously forecasts patient trajectories and serves diverse downstream risk-prediction tasks without per-task fine-tuning—remains an open challenge. Existing JEPA frameworks either discard the predictor after pretraining (I-JEPA, V-JEPA) or train it on a frozen pretrained encoder (V-JEPA 2-AC), leaving the encoder unaware of the rollout signal that the retained predictor must use at inference; co-training the encoder and predictor under a shared JEPA prediction objective would supply this grounding, but naïve co-training is unstable, with representation collapse and online/target drift causing autoregressive rollout to diverge. Clin-JEPA’s five-phase pretraining curriculum—predictor warmup, joint refinement, EMA target alignment, hard sync, and predictor finalization—addresses each failure mode by phase, stably co-training a Qwen3-8B-based encoder and a 92M-parameter latent trajectory predictor. On MIMIC-IV ICU data, three independent evaluations support the framework: (1) latent $\ell_{1}$ rollout drift uniquely *converges* (−15.7%) over 48-hour horizons while baselines and ablations diverge (+3% to +4951%); (2) the encoder learns a clinically discriminative latent geometry (deteriorating-patient cohorts displace 4.83× further than stable patients in latent space, vs ≤2.62× for baseline encoders); (3) a single backbone outperforms strong tabular and sequence baselines on multi-task downstream evaluation. Clin-JEPA achieves mean AUROC 0.851 on ICareFM EEP and 0.883 on 8 binary risk tasks (+0.038 and +0.041 vs baseline average).

## 1 Introduction

A patient’s stay in the intensive care unit (ICU) is a high-dimensional dynamical system: every hour brings new vital signs, laboratory values, and clinical interventions whose effects on patient state must be accounted for in both forecasting and risk prediction. Existing electronic-health-record (EHR) modelling approaches address only part of this problem. Token-autoregressive language models trained on tokenised EHR sequences [1–4] can generate plausible event trajectories, but treat the patient as a sequence of static text events and never explicitly model the underlying continuous physiological state. Per-task foundation models for the ICU [5] achieve strong task-level performance, but require per-task feature engineering and fine-tuning with no unified representation that supports trajectory simulation.

Recent latent world models in vision and robotics offer a promising direction. Joint-Embedding Predictive Architectures (JEPA) [6–8] pre-train representations by predicting masked or future content in a latent space rather than reconstructing pixels, and V-JEPA 2-AC [9] retains an action-conditioned predictor at inference to enable latent-space rollout for robotic planning. Adapting this paradigm to clinical EHR—where the analogous goal is a patient-state simulator that consumes free-text observations and interventions—is appealing but non-trivial.

Existing JEPA designs leave the inference-time-simulator use case open. I-JEPA [7] and V-JEPA [8] discard the predictor after pretraining, so only the encoder is available downstream and trajectory simulation is impossible. V-JEPA 2-AC [9] retains the predictor but trains it on a *frozen* pretrained encoder, leaving the encoder unaware of the rollout signal that the retained predictor must use at inference. Co-training the encoder and predictor under a shared JEPA prediction objective would simultaneously close both gaps—producing an encoder whose representations are dynamically grounded for the predictor, and a predictor that operates in a representation space jointly optimized for it. However, naïve co-training is unstable: the encoder is dragged toward representation collapse by an untrained predictor, and the predictor’s autoregressive rollout diverges as it accumulates errors in a moving target latent space.

**Figure 1:**
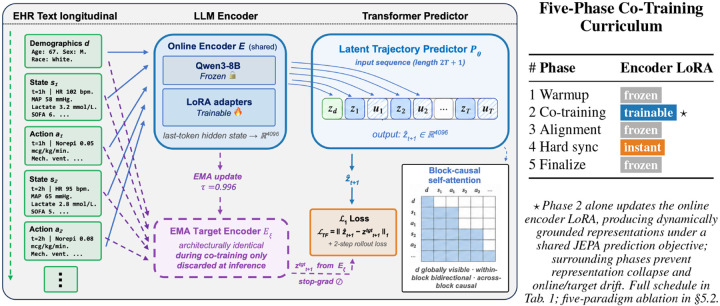
The Clin-JEPA framework. (*Left*) Three text inputs (demographics d, per-hour state $s_{t}$, action $a_{t}$) are processed by a single shared encoder E — Qwen3-8B base (frozen) with LoRA adapters (trainable) — producing 4096-dim latent embeddings via last-token extraction. The latent trajectory predictor $P_{\theta }$ is a block-causal Transformer that consumes the interleaved sequence $\left[z_{d},z_{1},u_{1},z_{2},u_{2},\ldots ,z_{T},u_{T}\right]$ and outputs ${\overset{ˆ}{z}}_{t+1}$. The EMA target encoder $E_{\xi }$ (dashed purple, trainingonly) provides a stop-gradient anchor for the $\ell_{1}$ teacher-forcing loss; at inference, $E_{\xi }$ is discarded and $E+P_{\theta }$ run autoregressively, chaining ${\overset{ˆ}{z}}_{t+h}$ back as next-step state input. (*Right*) Five-phase pretraining curriculum: only Phase 2 (⋆) updates the online encoder LoRA, with surrounding phases preventing the two failure modes that derail naïve co-training.

We present Clin-JEPA, a multi-phase co-training framework that closes this gap. Our contributions are: (1) a five-phase pretraining curriculum (predictor warmup, joint encoder–predictor refinement, EMA target alignment, hard sync, and predictor finalization) that stably co-trains a Qwen3-8B-based encoder with a retained latent trajectory predictor on MIMIC-IV ICU data [10]; (2) a text-based EHR representation that consumes raw clinical text and serves all downstream tasks from a single set of latent embeddings, requiring no missing-value imputation, no normalization, and no hand-engineered features; and (3) a three-axis empirical evaluation showing that Clin-JEPA uniquely converges over a 48-hour autoregressive horizon, learns a clinically discriminative latent geometry that separates deteriorating from stable patient cohorts, and outperforms strong tabular and sequence baselines on two standard ICU benchmarks. The framework is summarized in Figure 1.

## 2 Related Work

### Clinical foundation models for EHR.

Pretrained models for EHR span early encoder-only architectures such as Med-BERT [11] (structured ICD code sequences) and GatorTron [12] (clinical-text encoder), generative or autoregressive trajectory models [1, 2, 13, 14], and reasoning-enhanced or instruction-tuned LLMs for EHR analysis [3, 4, 15]. Multi-task ICU foundation models [5, 16, 17] achieve strong task-level performance through zero-shot in-context evaluation or per-task fine-tuning; recent empirical analysis [18] documents cross-institution transferability challenges. Across these architectures, none provides an explicit latent dynamical state representation suitable for autoregressive trajectory simulation.

### JEPA paradigm.

Joint-embedding predictive architectures (JEPA) [6] were realized for vision in I-JEPA [7] and V-JEPA [8], then extended with a retained action-conditioned predictor for robotic planning (V-JEPA 2-AC [9]), to language (LLM-JEPA [19]), and to vision-language (VL-JEPA [20]). The closest medical adaptation, SMB-Structure [21], jointly applies SFT and JEPA over masked future-token spans on longitudinal oncology EHR with an LLM encoder, but uses only the encoder’s embeddings for downstream tasks via linear probes.

### Latent world models for clinical decision support.

A parallel line of work introduces explicit latent world models for ICU and oncology decision support. medDreamer [22] adopts a Dreamer-style [23] RSSM trained from scratch with a discrete action space for sepsis policy learning. Latent Physiology as Language [24] models patients as a continuous-time latent SDE with EHR events as control inputs, trained jointly with reconstruction, masked imputation, and intervention-aware rollout-consistency objectives. CLARITY [25] pairs a LoRA-adapted MRI foundation encoder with an action-conditioned latent predictor on brain-tumor and breast-cancer MRI cohorts. The Qazi et al. [26] survey reviews this emerging paradigm. None of the above jointly grounds an LLM encoder against the rollout signal of a retained latent predictor — the gap Clin-JEPA closes with a multi-phase co-training curriculum.

## 3 Clin-JEPA Framework

The Clin-JEPA framework comprises two deployed components illustrated in Figure 1: an LLM-based encoder that maps EHR text into a continuous latent space, and a Transformer that autoregressively predicts patient trajectories in that latent space. §3.1 introduces the problem formulation and encoder; §3.2 details the latent trajectory predictor.

### 3.1 Problem Formulation and Patient State Encoding

A patient’s ICU stay constitutes a longitudinal EHR sequence of clinical events—observations (vital signs, laboratory measurements, severity scores) and interventions (medications, ventilator settings, procedures). We model each stay over a 72-hour window discretized into one-hour bins (consistent with the 24–72 h analysis windows commonly used in ICU forecasting literature [27, 28]). This yields a sequence of state–action pairs of length T≤72 preceded by a static demographics descriptor d available at admission:

(1) $$𝒮=\left\{d,\left(s_{1},a_{1}\right),\left(s_{2},a_{2}\right),\ldots ,\left(s_{T},a_{T}\right)\right\},$$

where $s_{t}$ records all observations occurring during hour t in their original temporal order and $a_{t}$ enumerates the clinical interventions active during hour t.

We represent state $s_{t}$, action $a_{t}$, and demographics d as structured natural-language text fragments rather than fixed numerical vectors. This unifies heterogeneous EHR signals (vitals, labs, drugs, procedures) under a single text representation, leverages the LLM’s pretrained linguistic knowledge, and preserves human readability for clinical interpretation. The state text concatenates one hour’s clinical readings (such as vital signs, laboratory values, severity scores), e.g., “t=12h | Heart rate: 88 bpm. MAP: 62 mmHg. Lactate: 2.1 mmol/L. …”. The action text enumerates active interventions and doses, e.g., “t=12h ∣ Norepinephrine: 0.08 mcg/kg/min. Propofol: 40 mcg/kg/min. …”. Within each hour we preserve every individual reading in its original temporal order—including repeated measurements of the same variable (e.g., MAP sampled multiple times during resuscitation)—as a lossless serialization that the encoder’s self-attention parses for intra-hour dynamics; the latent trajectory predictor (§3.2) then composes these per-hour embeddings into inter-hour dynamics. This representation requires no value imputation, no normalization, and no modality-specific featurization: missing readings are simply absent from the state text, and numeric values appear in their natural clinical units.

Each of the three text inputs (state $s_{t}$, action $a_{t}$, and demographics d) is independently mapped to a 4096-dimensional latent embedding by our encoder E: a Qwen3-8B language model whose base weights are frozen and adapted via lightweight LoRA adapters. The same shared encoder processes state, action, and demographics text via independent forward passes, with the last-token hidden state taken as the embedding:

(2) $$z_{t}=E\left(s_{t}\right),\;u_{t}=E\left(a_{t}\right),\;z_{d}=E\left(d\right),\;z_{t},u_{t},z_{d}\in R^{4096}.$$

The LoRA adapters are first initialized via supervised next-token-prediction fine-tuning on per-hour state and action text, then refined jointly with the latent trajectory predictor (§3.2) under the multi-phase co-training curriculum (§4). LoRA configuration and training compute are reported in §5.1.

Clin-JEPA’s central objective is to predict the patient’s future latent trajectory over an H-hour horizon given past context and a proposed action sequence, autoregressively in the encoder’s $R^{4096}$ latent space:

(3) $${\overset{ˆ}{z}}_{t+h}=P_{\theta }\left({\tilde{z}}_{1:t+h-1},\;u_{1:t+h-1},\;z_{d}\right),\;h=1,\ldots ,H,$$

where ${\tilde{z}}_{j}=z_{j}$ for j≤t (encoded ground truth) and ${\tilde{z}}_{j}={\overset{ˆ}{z}}_{j}$ for j>t (the predictor’s own prior output, fed back at each rollout step), and $P_{\theta }$ is the latent trajectory predictor (§3.2). The encoded history $\left\{z_{1},\ldots ,z_{t}\right\}$ together with the autoregressive rollout ${\left\{{\overset{ˆ}{z}}_{t+h}\right\}}_{h=1}^{H}$ jointly serves as the patient-state representation for downstream clinical tasks.

### 3.2 Latent Trajectory Predictor

The latent trajectory predictor $P_{\theta }$ is a Transformer encoder (~92M parameters) that operates entirely in the 4096-dim encoder latent space. It consumes the demographics-state-action sequence $\left[z_{d},z_{1},u_{1},z_{2},u_{2},\ldots ,z_{T},u_{T}\right]$ of length 2T+1, where $z_{d}$ occupies position 0 as a global context token and $\left(z_{t},u_{t}\right)$ pairs occupy positions (2t-1,2t). Before entering the Transformer, each encoder embedding is projected from $R^{4096}$ to the predictor’s hidden dimension $R^{1024}$ by one of three modality-specific linear maps—one for state, one for action, one for demographics:

(4) $$h_{t}^{\left(s\right)}=W_{s}z_{t},\;h_{t}^{\left(a\right)}=W_{a}u_{t},\;h_{d}=W_{d}z_{d},\;W_{s},W_{a},W_{d}\in R^{1024\times 4096},$$

and a learned absolute positional embedding is added to $h_{t}^{\left(s\right)},h_{t}^{\left(a\right)},h_{d}$ at each position.

Self-attention is block-causal (Figure 1, mask inset): demographics is globally visible; tokens within a timestep block attend bidirectionally; across blocks attention is strictly causal. An output linear projection yields the absolute next-state prediction ${\overset{ˆ}{z}}_{t+1}\in R^{4096}$ from each state position.

At inference, $P_{\theta }$ is retained and rolls out autoregressively: each predicted ${\overset{ˆ}{z}}_{t+h}$ is fed back as the state input for step t+h+1, yielding the simulated trajectory ${\left\{{\overset{ˆ}{z}}_{t+h}\right\}}_{h=1}^{H}$ used for downstream clinical tasks (Eq. 3). Unlike token-LM autoregression, which samples a discrete token and feeds the sample back, our rollout feeds back the predicted continuous embedding directly—a deterministic conditionalmean composition that lets a single predictor cover all forecast horizons h=1,…,H with one shared model rather than training H horizon-specific predictors.

## 4 Multi-Phase Co-Training Pretraining

Building on the SFT-initialized encoder of §3.1, we jointly co-train the encoder LoRA adapters and the latent trajectory predictor under a shared JEPA prediction objective. This co-training is motivated by Clin-JEPA’s deployment regime: unlike I-JEPA [7] and V-JEPA [8], which discard the predictor after pretraining, our predictor is retained at inference and rolls out autoregressively to simulate patient trajectories. For these rollouts to produce clinically faithful dynamics, the encoder cannot only learn statically rich features (the regime image and video JEPA optimize for); it must learn representations that are *dynamically grounded* — organized so that the predictor can compose them into long autoregressive trajectories. Sharing a single JEPA prediction objective between encoder and predictor is what produces this grounding, and it is what distinguishes our framework from V-JEPA 2-AC [9], where the predictor is trained on a frozen pretrained encoder and the encoder never sees the rollout signal.

This co-training is, however, unstable in the shared latent space: a naïve implementation leads to two characteristic failure modes — *representation collapse* (the encoder degenerates to constant outputs the predictor can match trivially) and *online/target space drift* (the predictor learns to forecast in a moving target latent space and diverges under autoregressive rollout). §4.1 formalizes the co-training objective and the EMA target encoder used to keep prediction targets stable; §4.2 introduces the five-phase schedule that prevents each failure mode by phase.

### 4.1 Co-Training Objective

The predictor is trained to minimize the $\ell_{1}$ distance between its predicted next-state embedding and the target encoder’s embedding of the actual next state. Letting 𝒱 denote the set of valid (sample b, time t) pairs in a training batch, the teacher-forcing loss is

(5) $${ℒ}_{\text{TF}}=\frac{1}{\left|𝒱\right|}\sum_{(b,t)\in 𝒱}{\left\|{\overset{ˆ}{z}}_{t+1}^{\left(b\right)}-z_{t+1}^{\text{target},(b)}\right\|}_{1},$$

where $\|\cdot {\|}_{1}$ denotes the $\ell_{1}$ norm summed over the embedding’s 4096 dimensions. The total per-step training loss combines this with a short autoregressive rollout loss, $ℒ={ℒ}_{\text{TF}}+{ℒ}_{\text{roll}}$, where ${ℒ}_{\text{roll}}$ averages the same $\ell_{1}$ penalty over a 2-step autoregressive horizon (using the rollout in Eq. 3) to encourage stability under multi-step inference. Following prior JEPA work [7, 8], we use $\ell_{1}$ rather than MSE for robustness to heavy-tailed clinical laboratory distributions.

Following the JEPA family [7–9], we maintain a separate *target encoder* that is architecturally identical to the online encoder (frozen Qwen3-8B with LoRA adapters) and updated as an exponential moving average of the online encoder’s parameters: $\theta_{\text{target}}\leftarrow \tau \theta_{\text{target}}+(1-\tau )\theta_{\text{online}}$, applied at each optimizer step with momentum τ=0.996 (matching I-JEPA’s default). The target encoder serves only as a stop-gradient anchor for $z_{t+1}^{\text{target}}$, carries no projection head, and is discarded entirely at inference.

The rollout loss admits two regimes depending on whether the online and target encoders agree. *Native rollout* (used when online ≡ target) chains the predictor’s own output back as the next-step state input, mirroring inference behaviour. *Teacher-forced rollout* (used in phases where online ≠ target) instead substitutes the real online embedding at each rollout step; this prevents a space-mismatch failure where chained predictor outputs (trained to land in target space) would be fed back into a pipeline that expects online-space inputs.

### 4.2 Five-Phase Curriculum

Table 1 summarizes the five-phase schedule, which we describe in turn.

**Phase 1 (Warmup).** The predictor is trained alone against the frozen SFT-initialized encoder, warming up the cold predictor on initial trajectory dynamics before any encoder co-adaptation. Without this warmup, the cold predictor would, when the encoder unlocks in Phase 2, immediately exert a strong gradient pull dragging the encoder toward trivial constant embeddings — the representation-collapse failure mode. **Phase 2 (Co-training).** This is the central refinement step of the curriculum: the encoder LoRA adapters are unlocked and optimized together with the predictor under the same JEPA prediction objective, with teacher-forced rollout sidestepping the space mismatch described in §4.1. It is the only phase in which the predictor’s gradient signal reaches the encoder, and therefore the phase where the encoder’s representations actually become *dynamically grounded*. **Phase 3 (Alignment).** The online encoder is re-frozen and the EMA target smoothly catches up to it. This soft alignment serves as a buffer before the explicit hard sync in Phase 4, preventing an abrupt parameter jump that would destabilize the predictor. **Phase 4 (Hard sync).** An instantaneous parameter copy from online to target eliminates any residual mismatch before native rollout resumes. **Phase 5 (Finalize).** The predictor is trained on the now-stabilized encoder under native autoregressive rollout — the same regime as inference — using the remaining compute budget to fully converge the predictor on the refined encoder representations. Each phase prevents a specific failure mode that emerges if it is removed; §5.2 reports a five-paradigm ablation empirically validating each component.

Together, the co-training objective (§4.1) and five-phase curriculum (§4.2) constitute Clin-JEPA’s complete pretraining recipe; §5 characterizes the resulting model’s behavior across three independent evaluation axes.

## 5 Experiments

We evaluate Clin-JEPA on MIMIC-IV ICU [10] along three independent axes, in the order *train* → *diagnose* → *apply*: §5.2 *proves* the co-training paradigm is stable and converges, §5.3 *diagnoses* the latent geometry the encoder learns, and §5.4 *applies* the learned representation to downstream multi-task evaluation. All three axes independently support Clin-JEPA over prior JEPA-family designs, ablations of our own curriculum, and strong tabular and sequence baselines.

### 5.1 Setup

#### Datasets.

All experiments use MIMIC-IV ICU [10], with 84,497 stays from 64,874 unique patients. We split at the *patient* level (70/15/15 train/val/test): all ICU stays from a given patient are assigned to the same split, preventing patient-level data leakage between train, validation, and test. Per-hour state and action representations are constructed from MIMIC-IV’s raw tables and the official mimic-code derived concept tables; the complete list of source tables and observation/action features is provided in Appendix A. Each stay is windowed at 1-hour resolution following §3.1 $\left(T_{\text{max}}=72\right)$; stays exceeding 72 hours yield overlapping windows at stride 12 hours, totaling ~197K training windows.

#### Pretraining.

Clin-JEPA pretraining follows the five-phase curriculum of §4 (predictor warmup, co-training, alignment, hard sync, finalize), executed on 8× NVIDIA H200 GPUs for ~54 wall-clock hours (≈430 GPU-hours). Full optimizer and architecture hyperparameters are deferred to Appendix B.

#### Downstream baselines.

For downstream evaluation (§5.4), we compare against four standard strong baselines from the clinical-ML literature: Ridge regression, LightGBM [29], LSTM [30], and TCN [31], trained on raw clinical features.

#### Curriculum ablation variants.

For curriculum ablation (§5.2), we compare Clin-JEPA against four training-paradigm variants: V-JEPA 2-AC style [9] (random-mask JEPA followed by AC-predictor training on the frozen encoder), SFT baseline w/o JEPA refinement (encoder uses only the §3.1 SFT-initialized LoRA), Clin-JEPA w/o warmup (Phase 1 removed), and Clin-JEPA w/o alignment (Phases 3 and 4 removed). All five paradigms share the SFT-initialized encoder and the same 92M predictor architecture; they differ in the encoder training objective and in per-paradigm training budgets and termination criteria, detailed in Appendix C.

### 5.2 Co-Training Stability and Convergence

We measure training stability through two signals: *rollout drift* — the L1 distance between the predictor’s autoregressive output ${\overset{ˆ}{z}}_{C+h}$ and the encoder’s true forward output $z_{C+h}$ over a 48-hour horizon, normalized by the h=1 value; and *representation collapse* — the standard deviation of encoder output ($z_{\text{std}}$, a standard self-supervised representation-collapse indicator [7, 8]), tracking whether the encoder degenerates to constant outputs. Across the five training paradigms defined in §5.1, only Clin-JEPA produces a converging, non-collapsing latent trajectory.

#### Clin-JEPA uniquely converges over long horizons.

**Figure 2:**
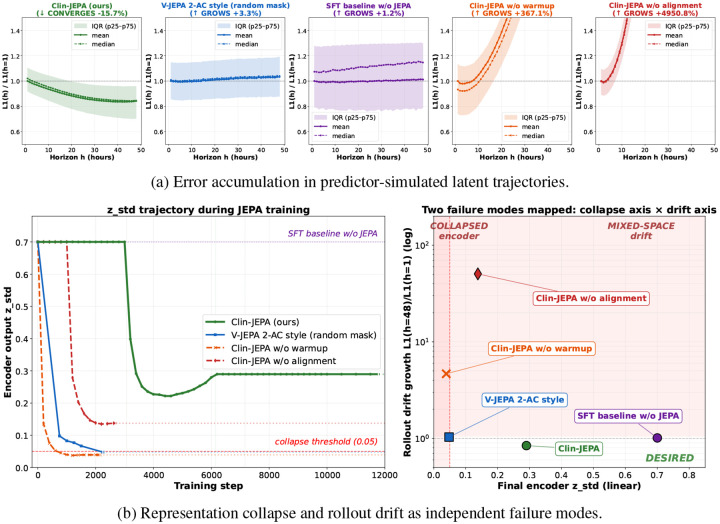
Co-training stability and convergence across five training paradigms. *(a)* Per-paradigm rollout error accumulation: mean drift trajectory with IQR shading (chosen over standard deviation for robustness to heavy-tailed drift in failure-mode paradigms) for each of the five training paradigms, evaluated at context C=24 on the held-out test split. Drift is the L1 distance between the predictor’s autoregressive output and the encoder’s true forward output, normalized by the h=1 value. *(b) Left*: $z_{\text{std}}$ trajectory during JEPA refinement, exposing representation collapse for paradigms that lack the warmup phase. *(b) Right*: joint distribution of final $z_{\text{std}}$ (x-axis) and drift accumulation (y-axis) across paradigms, showing collapse and drift are independent failure modes; only Clin-JEPA occupies the desired (high-$z_{\text{std}}$, low-drift) quadrant.

Over the 48-hour rollout horizon, Clin-JEPA’s mean rollout error *decreases* by −15.7% relative to its h=1 value (Figure 2a), while V-JEPA 2-AC style is essentially flat (+3.4%) and the SFT baseline shows modest divergence (+6.8% median). Both ablations diverge catastrophically: removing warmup yields +367% drift accumulation, and removing alignment yields +4951%. The convergence pattern in Clin-JEPA reflects an underlying clinical reality: most ICU patients in our test set transition toward a relatively stable physiological attractor near end-of-stay (recovery to baseline, transition to comfort care, or pre-discharge stable state), while early hours are dominated by acute decompensation and rapid intervention response. A predictor that has learned the underlying clinical dynamics should therefore predict late-horizon states more accurately than early-horizon ones — exactly the pattern Clin-JEPA exhibits. Baselines and ablations that fail to learn the dynamics either drift or remain flat across horizon.

#### Every component of the curriculum is necessary.

*Without warmup* (V-JEPA 2-AC style and Clin-JEPA w/o warmup), the encoder collapses during early JEPA refinement: $z_{\text{std}}$ drops below 0.05 — about 7% of the SFT-pretrained baseline $\left(z_{\text{std}}=0.700\right)$, our operational threshold for representation collapse (Appendix C; Figure 2b, left). The two warmup-protected variants (Clin-JEPA and Clin-JEPA w/o alignment) maintain healthy $z_{\text{std}}$ throughout training. *Without alignment* (Clin-JEPA w/o alignment), the predictor instead learns to map online-space contexts to target-space outputs, then chains those outputs back as online-space inputs at the next rollout step, compounding error across the rollout horizon. Together, the drift evidence in Figure 2a and the collapse evidence in Figure 2b (left) expose two independent failure modes (Figure 2b, right): only Clin-JEPA, with both warmup and alignment, occupies the desired (high-$z_{\text{std}}$, low-drift) quadrant.

### 5.3 Latent Geometry Diagnosis

**Figure 3:**
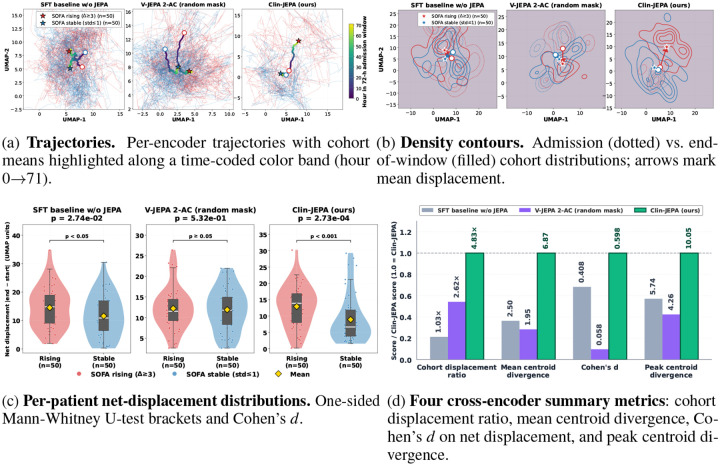
Latent-geometry diagnosis: deteriorating-vs-stable cohort discrimination across three encoder variants. Each subfigure (a–d) shows one analysis view comparing the same three encoders. Only Clin-JEPA (**ours**) produces a clinically discriminative latent geometry.

§5.2 established that Clin-JEPA trains stably; we now ask what its encoder *learned*. Following the JEPA family’s standard evaluation axis [7–9], we probe encoder representation geometry directly — the upstream bottleneck for our retained-predictor design (§3.2). From the test set, we identify two extreme phenotypes — 50 *deteriorating* patients (progressive organ failure: △SOFA≥3 over 72 h) and 50 *stable* patients (constant disease severity) — and project each patient’s per-hour 4096-dim embeddings through per-encoder UMAP fits (Figure 3). Across all four analysis views, only Clin-JEPA produces a clinically discriminative latent geometry.

#### Visual separation is immediately apparent.

Figure 3a traces 100 individual patient trajectories per encoder over the 72-hour window: under Clin-JEPA, the deteriorating and stable cohort means visibly diverge as the window unfolds, while V-JEPA 2-AC style keeps both cohort means tightly clustered together and SFT drifts them apart without recognizable cohort-level structure. Figure 3b makes the same separation distributional: Clin-JEPA’s admission contours (dotted) and end-of-window contours (filled) for the two cohorts barely overlap, with markedly asymmetric cohort-mean displacement arrows; the two baseline encoders show extensive admission/end overlap and near-symmetric arrow lengths. Quantification (panels c–d) confirms what is visible by eye.

#### The deeper signature: stable patients staying put.

The diagnostic of a clinically grounded encoder is not how far deteriorating patients move, but how steady stable patients are kept — a naïve encoder would let every patient’s latent representation drift from accumulated noise alone. The cohort displacement ratio — how far the deteriorating cohort centroid moves over 72 hours, divided by how far the stable cohort centroid moves — is **4.83**× for Clin-JEPA, **2.62**× for V-JEPA 2-AC style, and **1.03**× for SFT (Figure 3d). Clin-JEPA barely moves stable patients while letting deteriorating patients traverse a long arc; SFT drifts both cohorts almost equally, suggesting its latent dynamics are dominated by representation noise rather than learned clinical structure. Per-patient net-displacement separation reaches Cohen’s d=0.598 (medium-large effect, $p=2.7\times 10^{-4}$) for Clin-JEPA, versus d=0.058 (n.s., p=0.53) for V-JEPA 2-AC style and d=0.408 (small-medium, p=0.027) for SFT (Figure 3c). Cohen’s d being standardized confirms the ~10× gap over V-JEPA 2-AC is real, not a UMAP scale artefact.

#### Discriminative power grows with observation horizon.

Clin-JEPA’s two cohort centroids reach maximum divergence of 10.05 UMAP units at hour 66 with a mean of 6.87 over the 72-hour window — the encoder distinguishes deterioration from stability *most clearly near the end* of the window. This temporal pattern mirrors the terminal-state convergence observed in §5.2: both reflect that Clin-JEPA’s encoder has internalized the slow, integrative timescale of ICU progression. V-JEPA 2-AC style instead peaks early (hour 27, divergence 4.26) and converges back; SFT drifts apart slowly without clear progression (mean 2.50). V-JEPA 2-AC’s early-peak-then-collapse pattern has a structural cause: its bidirectional masked-reconstruction pretraining never sees the rollout signal, removing the incentive to preserve cohort separation under long-horizon AR composition. Clin-JEPA’s curriculum is the only paradigm we tested whose discriminative power *grows* with horizon—the property an inference-time autoregressive simulator requires, quantified downstream in §5.4.

### 5.4 Downstream Multi-Task Evaluation

#### Setup.

We *apply* the deployed Clin-JEPA model (encoder + retained predictor) to two complementary clinical task families. *Track 1 — ICareFM Early Event Prediction (EEP)* [5] comprises 7 multi-criteria event-prediction tasks (circulatory, respiratory, kidney, liver, hyperglycemia, sepsis-3, decompensation) at horizons of 8–48 hours. *Track 2 — a stay-level clinical risk benchmark* [1–4] comprises 8 admission-anchored binary outcomes (six mortality variants, prolonged-stay-7d, and sepsis-ever) on 10,346 test stays. Both tracks fix the encoder context length to C=24 hours. Whereas the four baselines (§5.1) are retrained per-task on hand-engineered features, Clin-JEPA’s deployed model serves all 15 tasks from a single set of latent embeddings without per-task fine-tuning.

#### Probe.

For each task, a shallow MLP probe (one hidden layer with ReLU) is trained on two feature configurations: a *history-only* variant $z_{\text{hist}}$ that pools the encoder’s embeddings of the 24-hour context (state, action, and statics), and a *history-plus-future* variant $z_{\text{full}}=z_{\text{hist}}⊕z_{\text{fut}}$, where $z_{\text{fut}}$ pools the encoder’s embeddings of the predictor’s autoregressive rollout over the remaining trajectory. The same probe architecture is used across all encoder variants and both task tracks for fair comparison.

#### Clin-JEPA leads both tracks.

**Figure 4:**
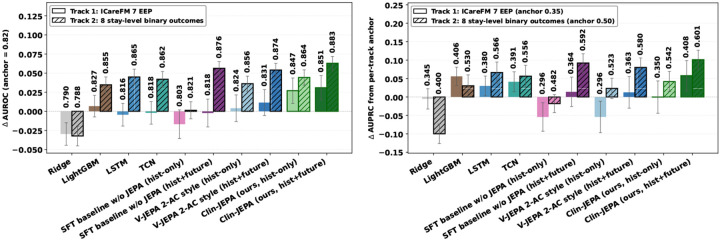
Downstream multi-task evaluation across two clinical benchmarks. Mean AUROC (*left*) and AUPRC (*right*) per method; paired bars are *Track 1 ICareFM EEP* (solid) and *Track 2 stay-level binary risk* (hatched). Clin-JEPA (**ours**) with $z_{\text{full}}$ leads AUROC on both tracks and AUPRC on Track 2; Track 1 AUPRC ties the strongest baseline.

On Track 1 (Figure 4), Clin-JEPA’s $z_{\text{full}}$ reaches mean AUROC **0.851**, exceeding the strongest baseline LightGBM (0.827) by +0.024 and both encoder ablations (V-JEPA 2-AC 0.831, SFT 0.818). On Track 2 it reaches **0.883**, beating LSTM (0.865) by +0.018. Track 2 AUPRC follows the same ordering (**0.601** vs. 0.566, +0.035); Track 1 AUPRC ties at the mean (0.408 vs. 0.406)—the AUROC–AUPRC divergence reflects LightGBM’s strength on low-base-rate threshold tasks. Per-task tables and 95% bootstrap CIs in Appendix D.

#### The retained predictor adds value.

The $z_{\text{hist}}\to z_{\text{full}}$ transition isolates the predictor’s contribution: on Track 2, lift is +0.019 mean AUROC with Clin-JEPA winning **all 8 of 8 outcomes**—confirming retained-predictor value beyond the §5.2 latent-rollout metric.

#### Where the representation pays off.

Clin-JEPA’s gains concentrate on tasks that require composing heterogeneous trajectory information into a coherent clinical assessment: kidney injury (+0.111 AUROC vs. LightGBM), sepsis-3 (+0.057), and decompensation (+0.189) on Track 1, and prolonged-stay-7d (+0.087 vs. LSTM) and sepsis-ever (+0.068) on Track 2. On simpler threshold-detection or acuity-driven tasks, feature-engineered baselines already extract close-to-sufficient signal from raw vital trends, and Clin-JEPA matches them within bootstrap CI rather than dominating. The pattern is systematic: gains where temporal composition matters, ties where it does not.

## 6 Conclusion

We presented Clin-JEPA, a multi-phase co-training framework for joint-embedding predictive pretraining on EHR patient trajectories. Our curriculum closes the gap left by prior JEPA designs that either discard the predictor or train it on a frozen encoder. To our knowledge, this is the first framework where a single clinical backbone delivers stable autoregressive rollout, clinically discriminative latent geometry, and competitive multi-task downstream performance from one set of latent embeddings.

## Figures and Tables

**Table 1:** The five-phase co-training curriculum; per-component ablation in §5.2. ≡ denotes parameter equality between online and target encoders.

Phase	Encoder LoRA	Target encoder	Rollout regime	Purpose of this phase
1. Warmup	frozen	online ≡ target (init)	Native	Predictor warmstart on SFT-initialized encoder
2. Co-training	trainable	EMA target, slow	Teacher-forced	Encoder + predictor jointly refined under stable target
3. Alignment	frozen	EMA chases online	Teacher-forced	Target encoder smoothly catches up to online
4. Hard sync	— (instant)	target ← online	—	Eliminate residual online-target mismatch
5. Finalize	frozen	online ≡ target (post-sync)	Native	Extended predictor training to convergence on stable encoder

## Appendix

### A Source tables and feature inventory

This appendix documents the MIMIC-IV [10] source tables and the per-hour features used to construct the encoder’s state and action text inputs (§3.1). Source tables prefixed concepts.* are official mimic-code derived concept tables; remaining sources (inputevents, prescriptions, procedureevents, …) are MIMIC-IV raw tables.

#### Inclusion criteria.

ICU stays included in the cohort satisfy: age ≥ 18, ICU length of stay ≥ 6 hours, and ≥ 1 observation event. Trajectory windows are capped at $T_{\text{max}}=72$ hours (1-hour discretization); stays exceeding 72 hours yield overlapping windows at stride 12 hours.

**Table 2:** Observation features (patient state) extracted from MIMIC-IV. Source tables prefixed concepts.* are official mimic-code derived concepts; raw tables are from MIMIC-IV directly.

Domain	Source table	Category	Variables
vitalsign	concepts.measurement.vitalsign	vitals	heart_rate, sbp, dbp, mbp, sbp_ni, dbp_ni, mbp_ ni, resp_rate, temperature, spo2, glucose
bg	concepts.measurement.bg	blood_gas	so2, po2, pco2, fio2, fio2_chartevents, ph, baseexcess, bicarbonate, totalco2, hematocrit, hemoglobin, chloride, calcium, potassium, sodium, lactate, glucose, aado2, aado2_calc, pao2fio2ratio
chemistry	concepts.measurement.chemistry	labs	albumin, globulin, total_protein, aniongap, bicarbonate, bun, calcium, chloride, creatinine, glucose, sodium, potassium
complete_blood_ count	concepts.measurement.complete_blood_count	labs	hematocrit, hemoglobin, mch, mchc, mcv, platelet, rbc, rdw, rdwsd, wbc
coagulation	concepts.measurement.coagulation	labs	d_dimer, fibrinogen, thrombin, inr, pt, ptt
enzyme	concepts.measurement.enzyme	labs	alt, ast, alp, ggt, ld_ldh, amylase, ck_cpk, ck_mb, bilirubin_total, bilirubin_direct, bilirubin_ indirect
inflammation	concepts.measurement.inflammation	labs	crp
cardiac_marker	concepts.measurement.cardiac_marker	labs	troponin_t, ck_mb, ntprobnp
gcs	concepts.measurement.gcs	assessment	gcs, gcs_motor, gcs_verbal, gcs_eyes, gcs_ unable
urine_output	concepts.measurement.urine_output	output	urineoutput
urine_output_rate	concepts.measurement.urine_output_rate	output	uo, urineoutput_6hr, urineoutput_12hr, urineoutput_24hr, uo_mlkghr_6hr, uo_mlkghr_ 12hr, uo_mlkghr_ 24hr, weight
kdigo_stages	concepts.organfailure.kdigo_stages	organ_failure	creat, creat_low_past_7day, creat_low_past_ 48hr, aki_stage_creat, uo_rt_6hr, uo_rt_12hr, uo_ rt_24hr, aki_stage_uo, aki_stage_crrt, aki_stage, aki_stage_smoothed
oxygen_delivery	concepts.measurement.oxygen_delivery	vitals	o2_flow, o2_flow_additional, o2_delivery_ device_1
blood_differential	concepts.measurement.blood_differential	labs	wbc, neutrophils_abs, lymphocytes_abs, monocytes_abs, eosinophils_abs, basophils_abs, bands, immature_granulocytes, nrbc
height	concepts.measurement.height	vitals	height
weight	concepts.demographics.weight_durations	vitals	weight, weight_type
sofa	concepts.score.sofa	score	sofa_24hours, respiration_24hours, coagulation_ 24hours, liver_24hours, cardiovascular_24hours, cns_24hours, renal_24hours
rhythm	concepts.measurement.rhythm	vitals	heart_rhythm, ectopy_type, ectopy_frequency

**Table 3:** Action features (clinical interventions) extracted from MIMIC-IV. Source tables prefixed concepts.* are official mimic-code derived concepts; inputevents, prescriptions, etc. are MIMIC-IV raw tables.

Domain	Source table	Category	Variables
vasoactive_agent	concepts.medication.vasoactive_agent	vasopressor	dopamine, epinephrine, norepinephrine, phenylephrine, vasopressin, dobutamine, milrinone
norepinephrine_ equivalent_dose	concepts.medication.norepinephrine_equivalent_ dose	vasopressor	norepinephrine_equivalent_dose
antibiotic	concepts.medication.antibiotic	antibiotic	antibiotic, route
ventilation	concepts.treatment.ventilation	ventilation	ventilation_status
ventilator_setting	concepts.measurement.ventilator_setting	ventilation_settings	fio2, peep, plateau_pressure, minute_volume, flow_rate, tidal_volume_set, tidal_volume_observed, tidal_ volume_spontaneous, respiratory_rate_set, respiratory_ rate_total, respiratory_rate_spontaneous, ventilator_ mode, ventilator_mode_hamilton, ventilator_type
rrt	concepts.treatment.rrt	dialysis	dialysis_present, dialysis_active, dialysis_type
crrt	concepts.treatment.crrt	dialysis	crrt_mode, system_active, blood_flow, dialysate_rate, dialysate_fluid, replacement_rate, replacement_fluid, prefilter_replacement_rate, postfilter_replacement_rate, ultrafiltrate_output, hourly_patient_fluid_removal, current_goal, citrate, heparin_dose, heparin_ concentration, access_pressure, filter_pressure, effluent_ pressure, return_pressure, clots, clotted, clots_ increasing
invasive_line	concepts.treatment.invasive_line	procedure	line_type, line_site
neuroblock	concepts.medication.neuroblock	neuromuscular_ blocker	drug_rate, drug_amount
inputevents	icu.inputevents	fluid_med_input	IV fluids, infusions, and blood products (crystalloids, colloids, albumin, PRBC, FFP, platelets) plus continuously-infused drugs (vasoactives, sedatives, analgesics, insulin); 260+ distinct items
procedureevents	icu.procedureevents	procedure	ICU procedures and device-duration events (arterial and central lines, tubes, imaging, bronchoscopy, extubation); 150+ distinct items
prescriptions	hosp.prescriptions	medication_order	Inpatient medication orders across all routes (PO, IV, SC, PR); 1500+ distinct named drugs and fluids
emar	hosp.emar	medication_admin	Electronic medication administration records (drug administered, route, dose); 900+ distinct items

### B Training hyperparameters

Table 4 lists all hyperparameters used in Clin-JEPA pretraining (§4). The encoder LoRA is first SFT-initialized via per-hour next-token prediction on state and action text (§3.1), then refined jointly with the predictor under the five-phase curriculum (§4.2).

**Table 4:** Full hyperparameters for Clin-JEPA training pipeline.

Stage / component	Parameter	Value
Encoder (architecture)	Base model	Qwen3-8B (8.2B parameters, 4096-dim hidden)
	LoRA rank r	16
	LoRA α	32
	LoRA target modules	q_proj, k_proj, v_proj, o_proj
	Trainable parameters	~15.3M (0.19% of base)
	Embedding extraction	last-token hidden state (4096-dim)
	Max sequence length	4096 tokens
	Attention impl.	Flash Attention 2
Predictor (architecture)	Architecture	Transformer encoder, pre-norm GELU
	Layers	6
	Hidden dimension	1024
	Attention heads	8
	FFN dimension	4096
	FFN dropout	0.15
	Attention dropout	0.10
	Positional encoding	learned absolute, max sequence length 149
	Output mode	absolute (not residual)
	Total parameters	92.5M
SFT initialization	Optimizer	AdamW
	Learning rate	1e-4
	LR schedule	cosine
	Epochs	1
	Sample format	per-(stay, hour) state OR action text (independent samples)
JEPA pretraining (curriculum)	Optimizer	AdamW
	Encoder learning rate	5e-5
	Predictor learning rate	5e-4
	LR schedule	cosine, 2% warmup over total steps
	Weight decay	0.04
	Gradient clip (encoder)	0.5
	Gradient clip (predictor)	1.0
	Effective batch	64 trajectory windows per step (8 GPUs × 8 per-GPU)
	Total optimizer steps	~11,776 (≈3.83 epochs over 197K-window train set)
	Phase schedule	Phase 1 (warmup): 3072 steps; Phase 2 (co-training): 3072; Phase 3 (alignment): 1024; Phase 4 (hard sync): instantaneous; Phase 5 (finalize): 4608
	Rollout horizon	2 steps
	Loss	$\ell_{1}$ teacher-forcing + 2-step rollout (Eq. 5)
	EMA momentum τ	0.996 (fixed in Phases 2 and 3)
	EMA precision	fp32 (online encoder bf16)
	Random seed	42
	Hardware	8× NVIDIA H200
	Wall-clock	~54 hours (8×H200, ≈430 GPU-hours)

### C Per-paradigm training protocol for the stability analysis

The five paradigms compared in Figure 2 (§5.2) share an identical SFT-initialized encoder, an identical 92M-parameter AC predictor architecture, identical optimizer settings (Appendix B), and an identical held-out evaluation split. They differ in the encoder training objective and in the training budget allocated to each run; because the paradigms therefore terminate at different training steps, we make these budgets and stopping criteria fully explicit here.

#### Training budgets.

The headline Clin-JEPA model follows the full five-phase curriculum for 11,776 optimizer steps (warmup 3072; co-training 3072; alignment 1024; finalize 4608). The two curriculum ablations (Clin-JEPA w/o warmup, w/o alignment) are run on the shorter 4608-step schedule of our original curriculum (≈0.39× the headline budget), with phase counts rescaled proportionally (warmup 1024 steps for w/o alignment; 0 for w/o warmup by construction). Extending each ablation to the full 11,776-step schedule would multiply the already-substantial multi-GPU-day cost of the five-way comparison; because the failure modes these ablations expose are early and irreversible (argued next), the additional compute yields no additional evidence.

#### Why the shorter ablation budget is sufficient and conservative.

Both failure modes are early, monotone, and irreversible. Representation collapse $\left(z_{\text{std}}\right)$ is determined within a few hundred steps of the encoder unlocking: the two no-warmup paradigms fall below the 0.05 collapse threshold within 800–2250 steps (V-JEPA 2-AC style [9] aborted at 0.048 while still falling, w/o warmup plateauing at 0.039), with no mechanism by which further training could restore a collapsed encoder. Online/target-space drift (the w/o-alignment failure) is a structural consequence of the predictor learning to map online-space contexts to target-space outputs in the absence of the alignment and hard-sync phases; this mismatch is fixed once native rollout begins and can only compound with further training. Allocating less training to the ablations is therefore conservative: additional steps would deepen collapse or amplify drift, never reverse them.

#### Per-paradigm termination.

(i) Clin-JEPA runs the full 11,776 steps. (ii) V-JEPA 2-AC style is stopped by an automatic early-abort that triggers when $z_{\text{std}}$ crosses our operational 0.05 collapse threshold (step ≈2250, $z_{\text{std}}=0.048$); $z_{\text{std}}$ is still falling monotonically at that point, and continued refinement would only drive the encoder further toward complete representation collapse. (iii) The SFT baseline performs no JEPA refinement, so its encoder is unchanged and its $z_{\text{std}}$ is constant at the SFT value 0.700 (the horizontal reference in Figure 2b, left). (iv) For Clin-JEPA w/o warmup we disable the auto-abort so that the collapse is observed in full rather than truncated at the threshold, and stop once both its $z_{\text{std}}$ (0.039, well below the threshold) and validation loss have plateaued. (v) Clin-JEPA w/o alignment is likewise run with the auto-abort disabled and stopped once its $z_{\text{std}}$ has settled at a stable, non-collapsed plateau (≈0.14) and its validation loss has flattened; its mixed-space failure mode is already fully instantiated, so further training would only deepen the rollout drift. Items (i)–(v) describe the encoder-side training traced in Figure 2b; the retained AC predictor is co-trained with the encoder for Clin-JEPA and its two ablations, and trained separately on the frozen encoder’s cached embeddings for the two-stage baselines (V-JEPA 2-AC style and SFT). In the latter case the predictor is trained until its validation loss stops improving — training halts after five consecutive evaluations show no further decrease (early stopping with patience 5), up to a maximum of 20 epochs.

#### Scope of the ablations.

The two curriculum ablations (w/o warmup, w/o alignment) are used only in the stability analysis of §5.2. They are not carried into the latent-geometry diagnosis (§5.3) or the downstream multi-task evaluation (§5.4), each of which compares only Clin-JEPA against the V-JEPA 2-AC style and SFT encoders (and, for §5.4, the tabular and sequence baselines) under an identical protocol. The ablations’ early termination therefore cannot affect any reported geometry or downstream result. Moreover, the downstream evaluation (§5.4) applies an identical MLP-probe protocol to every encoder, and the two-stage baselines’ AC predictors share Clin-JEPA’s predictor architecture and optimizer settings; the comparison is therefore apples-to-apples, with performance differences reflecting the encoder training paradigm rather than any evaluation or predictor-training asymmetry.

#### Collapse threshold.

We monitor representation collapse via $z_{\text{std}}$, the encoder’s *per-window temporal* standard deviation (the variation of its outputs across the hours of a single trajectory), and define collapse operationally as $z_{\text{std}}$ falling below 0.05 — ≈7% of the healthy SFT baseline $\left(z_{\text{std}}=0.700\right)$. A value near zero indicates the encoder has degenerated to near-constant outputs across time, eliminating the temporal structure the predictor must roll out; this applies the anti-collapse rationale of variance regularization [32] — keeping a standard-deviation statistic above a fixed floor to prevent constant outputs — along the *temporal* axis, rather than the cross-sample (per-dimension, across-batch) axis that VICReg itself regularizes. The two are distinct: an encoder may retain enough cross-sample variance to support static linear probes while having lost the within-trajectory temporal variance required for faithful autoregressive rollout — precisely the regime the V-JEPA 2-AC style baseline occupies (strong enough for the static downstream tasks of §5.4, yet dynamically degenerate in §5.2–§5.3). The threshold serves only as an early-abort signal and a visual reference; the qualitative conclusion (warmup is necessary to prevent collapse) is insensitive to its exact value, as both collapsing paradigms fall below 0.10 within 800 steps.

### D Per-task downstream results

Full per-task tables for the downstream evaluation in §5.4. Bootstrap 95% confidence intervals computed from $n_{\text{boot}}=500$ resamples clustered by stay. Bolded cells indicate the per-column maximum.

**Table 5:** Track 1: ICareFM 7 EEP tasks — AUROC (95% CI), C=24h. Higher is better. Best per task in bold.

Method	Circulatory 8h	Respiratory 24h	Kidney 48h	Liver 48h	Hyperglycemia 8h	Sepsis-3 8h	Decomp 24h	Mean
Ridge	0.955 _[0.948,0.961]_	0.783 _[0.764,0.800]_	0.696 _[0.679,0.712]_	0.948 _[0.938,0.956]_	0.647 _[0.633,0.660]_	0.693 _[0.669,0.721]_	0.809 _[0.795,0.823]_	0.790
LightGBM	**0.975**_[0.969,0.979]_	**0.794**_[0.776,0.812]_	0.686 _[0.670,0.702]_	**0.960**_[0.951,0.967]_	**0.846**_[0.838,0.853]_	0.723 _[0.690,0.754]_	0.804 _[0.791,0.816]_	0.827
LSTM	0.965 _[0.959,0.970]_	0.791 _[0.773,0.809]_	0.718 _[0.702,0.736]_	0.953 _[0.943,0.961]_	0.681 _[0.668,0.695]_	0.738 _[0.709,0.766]_	0.862 _[0.848,0.873]_	0.816
TCN	0.964 _[0.957,0.969]_	0.790 _[0.770,0.808]_	0.723 _[0.705,0.738]_	0.952 _[0.943,0.960]_	0.676 _[0.663,0.689]_	0.752 _[0.724,0.782]_	0.869 _[0.856,0.880]_	0.818
SFT w/o JEPA (hist-only)	0.881 _[0.863,0.897]_	0.593 _[0.565,0.621]_	0.790 _[0.776,0.805]_	0.889 _[0.869,0.904]_	0.749 _[0.735,0.761]_	0.744 _[0.712,0.777]_	0.976 _[0.968,0.983]_	0.803
SFT w/o JEPA (hist+fut)	0.882 _[0.864,0.898]_	0.729 _[0.707,0.752]_	0.802 _[0.788,0.816]_	0.886 _[0.867,0.902]_	0.743 _[0.729,0.756]_	0.691 _[0.653,0.727]_	0.991 _[0.984,0.996]_	0.818
V-JEPA 2-AC (hist-only)	0.877 _[0.858,0.892]_	0.755 _[0.731,0.777]_	0.793 _[0.777,0.807]_	0.897 _[0.879,0.911]_	0.726 _[0.712,0.740]_	0.747 _[0.717,0.780]_	0.974 _[0.969,0.980]_	0.824
V-JEPA 2-AC (hist+fut)	0.876 _[0.858,0.892]_	0.759 _[0.737,0.782]_	**0.810**_[0.797,0.824]_	0.900 _[0.884,0.914]_	0.724 _[0.709,0.738]_	0.757 _[0.724,0.791]_	0.993 _[0.990,0.996]_	0.831
**Clin-JEPA (hist-only)**	0.894 _[0.878,0.910]_	0.773 _[0.750,0.793]_	0.787 _[0.773,0.803]_	0.911 _[0.896,0.924]_	0.811 _[0.798,0.822]_	0.776 _[0.744,0.808]_	0.976 _[0.970,0.982]_	0.847
**Clin-JEPA (hist+fut)**	0.894 _[0.878,0.908]_	0.773 _[0.750,0.792]_	0.797 _[0.783,0.813]_	0.911 _[0.895,0.924]_	0.809 _[0.797,0.821]_	**0.780**_[0.748,0.810]_	**0.993**_[0.989,0.996]_	**0.851**

**Table 6:** Track 1 (cont.): ICareFM 7 EEP tasks — AUPRC (95% CI), C=24h. Higher is better. Best per task in bold.

Method	Circulatory 8h	Respiratory 24h	Kidney 48h	Liver 48h	Hyperglycemia 8h	Sepsis-3 8h	Decomp 24h	Mean
Ridge	0.719 _[0.679,0.752]_	0.278 _[0.244,0.315]_	0.222 _[0.198,0.244]_	0.709 _[0.652,0.751]_	0.251 _[0.234,0.272]_	0.022 _[0.018,0.027]_	0.214 _[0.191,0.240]_	0.345
LightGBM	**0.788**_[0.754,0.817]_	**0.311**_[0.273,0.352]_	0.187 _[0.166,0.208]_	**0.803**_[0.763,0.835]_	**0.570**_[0.548,0.591]_	0.026 _[0.020,0.033]_	0.159 _[0.142,0.178]_	0.406
LSTM	0.758 _[0.723,0.788]_	0.286 _[0.251,0.322]_	0.221 _[0.196,0.243]_	0.774 _[0.728,0.809]_	0.284 _[0.262,0.308]_	0.027 _[0.022,0.035]_	0.310 _[0.285,0.337]_	0.380
TCN	0.759 _[0.722,0.787]_	0.299 _[0.263,0.338]_	0.241 _[0.216,0.264]_	0.778 _[0.730,0.813]_	0.281 _[0.259,0.304]_	0.028 _[0.022,0.035]_	0.354 _[0.329,0.380]_	0.391
SFT w/o JEPA (hist-only)	0.342 _[0.291,0.391]_	0.121 _[0.101,0.151]_	0.285 _[0.262,0.310]_	0.543 _[0.481,0.600]_	0.387 _[0.358,0.412]_	0.043 _[0.036,0.057]_	0.349 _[0.280,0.429]_	0.296
SFT w/o JEPA (hist+fut)	0.338 _[0.284,0.390]_	0.217 _[0.181,0.255]_	0.301 _[0.277,0.326]_	0.526 _[0.460,0.588]_	0.376 _[0.347,0.401]_	0.038 _[0.029,0.056]_	0.752 _[0.689,0.809]_	0.364
V-JEPA 2-AC (hist-only)	0.325 _[0.274,0.378]_	0.236 _[0.195,0.277]_	0.295 _[0.273,0.323]_	0.547 _[0.476,0.615]_	0.352 _[0.325,0.377]_	0.050 _[0.039,0.074]_	0.267 _[0.206,0.341]_	0.296
V-JEPA 2-AC (hist+fut)	0.316 _[0.265,0.368]_	0.243 _[0.202,0.280]_	**0.324**_[0.299,0.352]_	0.549 _[0.476,0.621]_	0.350 _[0.324,0.376]_	0.052 _[0.040,0.076]_	0.705 _[0.635,0.766]_	0.363
**Clin-JEPA (hist-only)**	0.370 _[0.314,0.430]_	0.254 _[0.215,0.293]_	0.277 _[0.255,0.303]_	0.644 _[0.586,0.702]_	0.491 _[0.461,0.518]_	**0.060**_[0.044,0.088]_	0.352 _[0.281,0.436]_	0.350
**Clin-JEPA (hist+fut)**	0.368 _[0.317,0.427]_	0.258 _[0.217,0.296]_	0.285 _[0.263,0.314]_	0.635 _[0.573,0.694]_	0.489 _[0.460,0.515]_	0.059 _[0.044,0.086]_	**0.759**_[0.700,0.815]_	**0.408**

**Table 7:** Track 2: 8 stay-level binary outcomes — AUROC (95% CI), C=24h, admission cohort. Higher is better. Best per task in bold.

Method	Hosp. mort.	True ICU mort.	Mort 7d	Mort 14d	Mort 30d	Mort 90d	Prolong. stay	Sepsis ever	Mean
Ridge	0.802 _[0.789,0.816]_	0.828 _[0.815,0.844]_	0.800 _[0.784,0.817]_	0.795 _[0.782,0.810]_	0.783 _[0.771,0.797]_	0.779 _[0.768,0.789]_	0.741 _[0.727,0.754]_	0.774 _[0.767,0.782]_	0.788
LightGBM	0.866 _[0.855,0.876]_	0.897 _[0.886,0.908]_	0.882 _[0.870,0.895]_	0.860 _[0.848,0.871]_	0.843 _[0.833,0.853]_	0.824 _[0.815,0.833]_	0.822 _[0.812,0.834]_	0.842 _[0.835,0.849]_	0.855
LSTM	**0.883**_[0.871,0.892]_	0.907 _[0.896,0.917]_	**0.895**_[0.881,0.906]_	**0.877**_[0.866,0.887]_	**0.861**_[0.850,0.871]_	**0.844**_[0.834,0.852]_	0.833 _[0.822,0.844]_	0.821 _[0.814,0.829]_	0.865
TCN	0.879 _[0.868,0.888]_	0.905 _[0.894,0.915]_	0.894 _[0.882,0.906]_	0.875 _[0.864,0.885]_	0.858 _[0.848,0.869]_	0.840 _[0.831,0.849]_	0.829 _[0.818,0.840]_	0.816 _[0.808,0.824]_	0.862
SFT w/o JEPA (hist-only)	0.850 _[0.839,0.860]_	0.889 _[0.878,0.898]_	0.602 _[0.582,0.623]_	0.861 _[0.851,0.871]_	0.845 _[0.834,0.855]_	0.828 _[0.818,0.837]_	0.838 _[0.827,0.848]_	0.858 _[0.850,0.865]_	0.821
SFT w/o JEPA (hist+fut)	0.869 _[0.858,0.878]_	0.906 _[0.896,0.914]_	0.888 _[0.875,0.900]_	0.867 _[0.857,0.877]_	0.854 _[0.844,0.864]_	0.835 _[0.826,0.843]_	0.915 _[0.910,0.921]_	0.877 _[0.869,0.883]_	0.876
V-JEPA 2-AC (hist-only)	0.859 _[0.848,0.869]_	0.893 _[0.883,0.903]_	0.872 _[0.859,0.883]_	0.859 _[0.849,0.869]_	0.844 _[0.833,0.854]_	0.827 _[0.817,0.836]_	0.839 _[0.828,0.849]_	0.856 _[0.848,0.863]_	0.856
V-JEPA 2-AC (hist+fut)	0.870 _[0.860,0.879]_	0.904 _[0.894,0.913]_	0.885 _[0.872,0.895]_	0.867 _[0.857,0.877]_	0.850 _[0.841,0.860]_	0.831 _[0.822,0.840]_	0.918 _[0.913,0.923]_	0.867 _[0.860,0.874]_	0.874
**Clin-JEPA (hist-only)**	0.867 _[0.856,0.877]_	0.896 _[0.885,0.906]_	0.877 _[0.864,0.889]_	0.865 _[0.855,0.876]_	0.849 _[0.839,0.859]_	0.834 _[0.823,0.842]_	0.842 _[0.830,0.852]_	0.884 _[0.878,0.890]_	0.864
**Clin-JEPA (hist+fut)**	0.875 _[0.865,0.884]_	**0.909**_[0.900,0.917]_	0.890 _[0.877,0.901]_	0.870 _[0.859,0.879]_	0.854 _[0.844,0.864]_	0.836 _[0.826,0.845]_	**0.919**_[0.913,0.925]_	**0.910**_[0.904,0.915]_	**0.883**

**Table 8:** Track 2 (cont.): 8 stay-level binary outcomes — AUPRC (95% CI), C=24h, admission cohort. Higher is better. Best per task in bold.

Method	Hosp. mort.	True ICU mort.	Mort 7d	Mort 14d	Mort 30d	Mort 90d	Prolong. stay	Sepsis ever	Mean
Ridge	0.359 _[0.331,0.392]_	0.328 _[0.295,0.364]_	0.265 _[0.236,0.302]_	0.327 _[0.301,0.359]_	0.394 _[0.370,0.423]_	0.464 _[0.443,0.489]_	0.310 _[0.290,0.334]_	0.755 _[0.744,0.767]_	0.400
LightGBM	0.499 _[0.463,0.529]_	0.502 _[0.463,0.540]_	0.434 _[0.395,0.474]_	0.480 _[0.444,0.515]_	0.523 _[0.494,0.552]_	0.557 _[0.533,0.582]_	0.423 _[0.401,0.452]_	0.825 _[0.814,0.835]_	0.530
LSTM	**0.549**_[0.517,0.582]_	0.537 _[0.504,0.578]_	0.490 _[0.452,0.532]_	0.523 _[0.492,0.556]_	0.567 _[0.539,0.94]_	**0.603**_[0.579,0.624]_	0.456 _[0.433,0.486]_	0.805 _[0.794,0.815]_	0.566
TCN	0.538 _[0.503,0.569]_	0.528 _[0.490,0.565]_	0.478 _[0.435,0.520]_	0.512 _[0.478,0.544]_	0.554 _[0.524,0.80]_	0.593 _[0.568,0.614]_	0.451 _[0.425,0.479]_	0.797 _[0.786,0.808]_	0.556
SFT w/o JEPA (hist-only)	0.444 _[0.416,0.479]_	0.450 _[0.416,0.487]_	0.099 _[0.088,0.114]_	0.477 _[0.446,0.506]_	0.528 _[0.501,0.554]_	0.564 _[0.541,0.588]_	0.442 _[0.418,0.470]_	0.853 _[0.843,0.863]_	0.482
SFT w/o JEPA (hist+fut)	0.526 _[0.497,0.554]_	0.542 _[0.513,0.575]_	0.542 _[0.506,0.574]_	**0.527**_[0.498,0.553]_	**0.569**_[0.545,0.590]_	0.587 _[0.565,0.609]_	0.573 _[0.549,0.601]_	0.873 _[0.864,0.881]_	0.592
V-JEPA 2-AC (hist-only)	0.472 _[0.444,0.506]_	0.465 _[0.429,0.501]_	0.402 _[0.364,0.439]_	0.463 _[0.432,0.492]_	0.520 _[0.494,0.549]_	0.561 _[0.539,0.585]_	0.452 _[0.428,0.482]_	0.850 _[0.840,0.860]_	0.523
V-JEPA 2-AC (hist+fut)	0.524 _[0.497,0.553]_	0.517 _[0.485,0.551]_	0.519 _[0.485,0.550]_	0.523 _[0.493,0.552]_	0.548 _[0.523,0.573]_	0.573 _[0.552,0.595]_	0.575 _[0.551,0.605]_	0.863 _[0.853,0.872]_	0.580
**Clin-JEPA (hist-only)**	0.495 _[0.464,0.526]_	0.485 _[0.450,0.519]_	0.433 _[0.393,0.468]_	0.479 _[0.446,0.509]_	0.534 _[0.507,0.560]_	0.573 _[0.550,0.596]_	0.459 _[0.434,0.487]_	0.878 _[0.869,0.886]_	0.542
**Clin-JEPA (hist+fut)**	0.539 _[0.511,0.568]_	**0.542**_[0.509,0.574]_	**0.549**_[0.513,0.580]_	0.521 _[0.489,0.550]_	0.559 _[0.533,0.583]_	0.590 _[0.567,0.612]_	**0.604**_[0.578,0.632]_	**0.906**_[0.898,0.912]_	**0.601**
